# The impact of bariatric surgery on the resolution of obstructive sleep apnoea

**DOI:** 10.1186/s13104-018-3484-5

**Published:** 2018-06-14

**Authors:** Toritseju Oluwafunmilayo Sillo, Simon Lloyd-Owen, Emma White, Karen Abolghasemi-Malekabadi, Penny Lock-Pullan, Muhammed Ali, Anthony Perry, Steven John Robinson, Martin Stuart Wadley

**Affiliations:** 1 Department of Upper Gastrointestinal and Bariatric Surgery, Worcestershire Acute Hospitals NHS Trust, Worcester, WR5 1DD UK; 2 Department of Thoracic Medicine, Worcestershire Acute Hospitals NHS Trust, Worcester, WR5 1DD UK

**Keywords:** Obesity, Sleep apnoea, obstructive, Bariatric surgery, Continuous positive airway pressure, Weight loss

## Abstract

**Objective:**

Obesity is associated with a high incidence of obstructive sleep apnoea (OSA). Bariatric surgery is postulated to lead to OSA resolution, but there is inconclusive evidence on its efficacy. We used objective measurements to determine the rate of resolution or improvement of OSA in patients who had bariatric procedures in our unit.

**Results:**

Data was analysed on all patients with OSA who underwent bariatric procedures [laparoscopic Roux-en-Y gastric bypass (LRYGB) and sleeve gastrectomy (LSG)] between June 2012 and September 2016 in our unit. 47 patients (26.7%) were diagnosed with OSA. Mean age was 48.5 years. 63.8% were female. 43 required nocturnal continuous positive airway pressure (CPAP) support. Procedures were LRYGB (n = 26) and LSG (n = 21). Mean excess weight loss was 56.1%. Mean start apnoea-hypopnoea index (AHI) on CPAP was 6.4 events/hr and end AHI was 1.4 events/h. 14 patients (32.6%) had complete OSA resolution and 12 (27.9%) showed improvement in pressure support requirements. We demonstrated that 55.3% of patients had resolution or improvement in OSA following bariatric surgery. However, there was a high rate of non-attendance of follow-up appointments. Future efforts will involve analysis of the reasons for this to ensure more robust monitoring.

## Introduction

Obstructive sleep apnoea (OSA) is strongly correlated with obesity. The incidence and severity of OSA rise with increase in body mass index (BMI) [1, 2]. Estimates of the prevalence of OSA in obese patients (BMI > 35 kg/m^2^) vary due to variations in diagnostic methodology and screening tools [3, 4]. It is characterised by recurrent upper airway obstruction during sleep leading to hypoxaemia and frequent awakening. Excessive daytime sleepiness is a cardinal symptom [5]. OSA is associated with adverse cardiovascular events and increased mortality from various causes including metabolic disease and cancer [6].

Current treatment includes behavioural changes, with the gold standard being the use of non-invasive continuous positive airway pressure (CPAP) devices [4, 7]. However while this is associated with improvements in health-related quality of life [4], it is not curative and there are often issues with concordance with therapy [8]. Recent trials have suggested minimal impact on objective outcomes such as adverse cardiovascular events [7, 9].

Bariatric surgery has been postulated to cause OSA resolution. A randomised controlled trial of laparoscopic adjustable gastric banding (LAGB) versus conventional weight loss methods in 60 obese patients with OSA found that while there was significantly greater excess weight loss (EWL) in the surgery group, both groups had significant decreases in the apnoea-hypopnoea index (AHI) [10]. Conversely, another randomised trial of CPAP versus LAGB in 49 patients showed a significant decrease in AHI in patients on CPAP compared with those who had LAGB, despite significantly greater weight loss in the LAGB group [11].

A systematic review by Sarkhosh et al. analysed pooled data from studies across 13,900 patients, and showed improvement or resolution in OSA after bariatric surgery in 88.5% of patients [12]. The effect was most marked with procedures with a malabsorptive mechanism (biliopancreatic diversion and gastric bypass). Another systematic review comparing bariatric surgery with non-surgical weight loss showed significant reduction in post-operative AHI in surgical compared with non-surgical patients [13]. However, most both studies combined data across heterogeneous studies to give pooled averages, with a high risk of bias.

The efficacy of bariatric surgery in the resolution of type two diabetes mellitus is conclusive [14], however data on its efficacy in OSA is less robust, due to differing study designs, low numbers of well-powered randomised trials, as well as insufficient concordance about screening methods and objective measurements of outcomes [15]. We aimed to determine the degree of improvement or resolution of OSA in our patient group using objective measures.

## Main text

### Methods

#### Patient selection

In the United Kingdom, adults with a BMI ≥ 40 kg/m^2^ or BMI ≥ 35 kg/m^2^ with defined comorbidities are eligible for referral to a Bariatric surgery service from primary care via a Tier 3 dietetic service, as per guidelines from the National Institute of Health and Care Excellence [16].

**Fig. 1:**
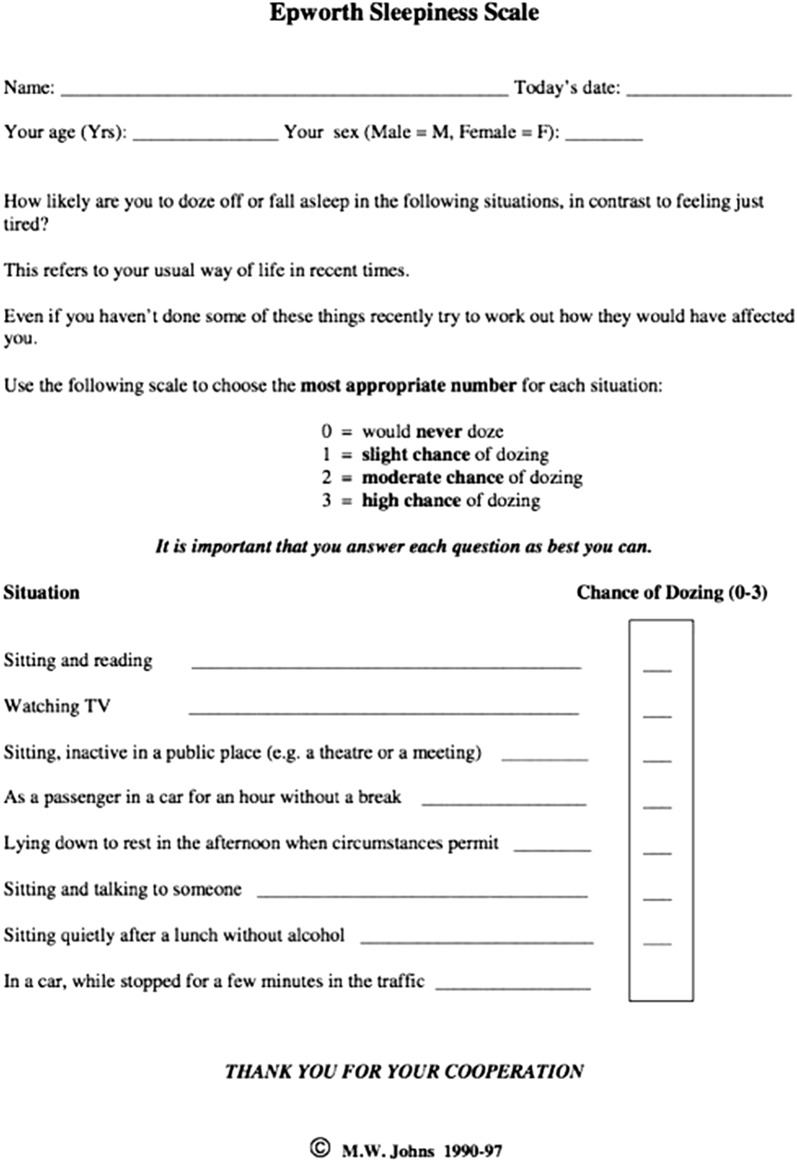
Epworth sleepiness scale [17]

The Bariatric service in Worcestershire was established in June 2012. All patients are screened for OSA pre-operatively using a combination of reported symptoms (particularly excessive daytime sleepiness and snoring), and Epworth Sleepiness scale (ESS) questionnaire (Fig. 1) [17]. An ESS of ≥ 10 triggers referral to the Clinical Investigations Unit for a home sleep study consisting of pulse oximetry, nasal flow, thoracic and abdominal movements and the measurement of movement and body position. The sleep studies are analysed by appropriately trained respiratory physiologists and an AHI score calculated. An AHI of > 10 triggers referral to the sleep clinic for further evaluation and commencement of nocturnal CPAP therapy if appropriate.

#### Data collection

A retrospective review of the prospectively maintained database of all patients between June 2012 and September 2016 who had bariatric surgery and were diagnosed with obstructive sleep apnoea was performed. Demographic details, start weight and BMI, end weight and BMI, % excess weight loss (EWL), pre- and post-operative AHI, requirement for CPAP therapy with start and end (if applicable) pressure settings were reviewed. Duration of follow-up and any losses to follow-up were also recorded.

### Statistical analysis

All values are expressed as mean ± standard deviation (SD) unless otherwise stated. %EWL was calculated as (start weight − end weight)/(start weight − ideal weight) × 100, with ideal weight given as that equivalent to a BMI of 25 kg/m^2^. Data was tested for parametricity using the Kolmogorov–Smirnov test. Differences between two groups with numerical data were tested using the two-sided student t test. Differences between groups with categorical data were tested using the χ^2^ test. Correlation between groups was tested using the Spearman rank correlation test. The statistical program used was GraphPad Prism version 5.

## Results

Of 176 patients in the database, 47 were formally diagnosed with OSA (26.7%). 63.8% of patients were female with a mean age of 48.5 years. The two procedures performed were laparoscopic Roux-en-Y gastric bypass (LRYGB) (n = 25) and laparoscopic sleeve gastrectomy (LSG) (n = 22). There were no significant differences in age, sex or start weight and BMI between the LRYGB and LSG groups. Patients with Type 2 diabetes mellitus were more likely to have the Roux-en-Y gastric bypass procedure (Table 1).

**Table 1:** Patient demographics

	LRYGB group (n = 25)	LGS group (n = 22)	p value	All patients (n = 47)
Age (years)	50.7 ± 10.2	46.0 ± 7.8	0.08^a^	48.5 ± 9.3
Sex (% female)	64.0	63.6	0.98^b^	63.8
Start weight (kg)	145.2 ± 22.6	139.5 ± 21.6	0.38^a^	142.5 ± 22.1
Start BMI (kg/m^2^)	52.1 ± 7.4	49.8 ± 7.8	0.31^a^	51.0 ± 7.6
% T2 diabetes	56.0	22.7	0.01^b^	42.6

^a^*t* test

^b^χ^2^ test

43 patients required nocturnal CPAP therapy. 4 patients were deemed not to require CPAP therapy after review in the sleep clinic. Of the 43 who were started on CPAP, the pre-operative AHI (whilst on CPAP therapy) and CPAP settings were available for 38 patients. There was a trend towards a higher start AHI in the LSG group (mean start AHI in the LRYGB group was 4.7 ± 4.7 events/h compared with 8.7 ± 7.1 events/h in the LSG group, p = 0.049). However, CPAP settings were similar for both groups (11.8 ± 3.0 cmH_2_O in the LRYGB group versus 10.7 ± 2.3 cmH_2_O in the LSG group, p = 019).

Mean duration of follow-up was 15.6 ± 10.6 months. 12 patients were lost to sleep clinic follow-up (25.5%); of these 10 did not attend scheduled appointments and 2 were non-adherent with the CPAP device and sleep clinic instructions.

At the end of the study period, mean excess weight loss was 56.2 ± 14.2% (average 39.6 kg weight loss) with a mean BMI of 36.4 ± 5.7 kg/m^2^. There was a significant decrease in the mean AHI post-operatively (p < 0.0001). 14 patients no longer required CPAP and were discharged from the sleep clinic (29.8%). A further 12 showed partial resolution with marked decreases in AHI and CPAP pressure settings, while 9 (19.1%) showed no objective improvement in settings (Table 2). At the end of the study period, 21 patients were still on CPAP therapy (44.7%) compared with 91.5% at the beginning.

**Table 2:** Objective measurements pre- and post-operatively

	Start (pre-operative)	End (post-operative)	p value
Weight (kg)	142.5 ± 22.1	102.9 ± 19.1	< 0.0001
BMI (kg/m^2^)	51.0 ± 7.6	36.4 ± 5.7	< 0.0001
AHI (events/hr)	6.4 ± 6.1	1.4 ± 1.7	< 0.0001
CPAP settings	11.1 ± 2.7	10.1 ± 2.7	0.17

Mean EWL was greater in the LRYGB group compared with the LSG group (p = 0.036) and end AHI also appeared lower in this group (p = 0.039). There were no other significant differences between the two groups

Correlation analyses were performed between EWL and end AHI and CPAP requirements, where available. There was no significant association found between EWL and end AHI or CPAP settings [Spearmank rank correlation co-efficient between EWL and end AHI r^2^ = − 0.28 (p = 0.17), between EWL and end CPAP r^2^ = − 0.24 (p = 0.20)].

## Discussion

In our study, 26.7% of patients who underwent weight loss surgery were diagnosed with OSA pre-operatively. Estimates of the prevalence of OSA in both obese and non-obese populations vary widely, thought mainly to arise due to differences in diagnostic criteria and screening tools used [4]. The incidence in the Wisconsin Sleep Study was estimated at 24 percent in men and 9% in women [1]. The current gold standard diagnostic tool is polysomnography in a sleep laboratory with an attendant technologist. However, this is expensive and cumbersome. Many screening tools have been developed to risk-stratify patients to determine suitability for further diagnostic testing, each with its limitations. The most well-established include the Epworth scale (the commonest tool in routine clinical practice) [17, 18], the STOP-Bang [19], Berlin [20] and Wisconsin sleep questionnaires [1]. They have all been validated in referral settings so their accuracy in a routine screening population is still untested [4].

Our study shows significant improvements in OSA after bariatric surgery, with 55.3% of patients demonstrating significant improvement or resolution using objective criteria. While this is a lower rate of resolution than in some of the published literature [12, 21, 22], it is based on objective measurements obtained from serial measurements during sleep clinic review rather than patient-reported symptoms as in much of the reported literature [12]. Although average pre-operative AHI readings whilst on CPAP were already below 10 events per hour (due to effective CPAP therapy), there were statistically significant decreases in AHI post-operatively. Based on these results, 30% of patients were successfully weaned off CPAP. Due to losses to follow-up, it is expected that the true proportion with complete resolution is higher.

The mechanisms through which this resolution occurs are still being elucidated. The “BRAVE” hypothesis (bile flow alteration, reduction of gastric size, anatomical gut rearrangement and altered flow of nutrients, vagal manipulation and **e**nteric gut hormone modulation) proposed by Ashrafian et al. [23] postulates that both weight-dependent and weight-independent metabolic effects play a role. We observed that improvements in AHI measurements and CPAP weaning occurred over several months. This is strikingly different from the rapid resolution of Type II diabetes typically observed after bariatric surgery (particularly RYGB). There did not appear to be a correlation between EWL and AHI or CPAP settings in this study, but statistical analysis was hampered by the small sample sizes due to losses to follow-up. In the future, anatomical measurements (such as neck size) and measurement of various hormone profiles will give a clearer understanding of the mechanisms and trajectory of resolution over time, as well as its link with excess weight loss.

## Limitations

Our primary screening tool was the Epworth scale, with a threshold for referral for a sleep study set at 10 or more on this scale. We acknowledge the potential risk that some patients with OSA, but no symptoms of excessive sleepiness, were not tested. The cost-effectiveness of routine sleep studies on all patients who present for Bariatric surgery is the subject of debate [4, 18].

There was a relatively high rate of non-attendance of follow-up sleep clinic sessions. Non-adherence to CPAP therapy is common in patients with OSA [24], with an estimated 46–83% of patients non-adherent with their CPAP device [25]. Many people with OSA are asymptomatic [26] therefore may not subjectively feel any benefit following treatment. Our patients report subjective improvements in sleep-apnoea related-symptoms after Bariatric surgery, tied to improvements in other co-morbidities. Some of these patients who subsequently attended Bariatric clinic follow-up had voluntarily ceased CPAP therapy. However, this needs to be correlated with objective measures. We are working to integrate the sleep clinic follow-up with the Bariatric service to ensure better adherence to follow-up and robustness of future data collection.

In summary, this study shows a clear benefit of Bariatric surgery to a group of patients with an established obesity-associated metabolic disorder with associated morbidity and mortality and costs from ongoing therapy. This adds to the body of literature supporting the role of Bariatric surgery in treating sleep apnoea and we believe that it should be routinely recommended for curative benefit in this patient group.

## Abbreviations

AHI: apnoea-hypopnoea index
BMI: body mass index
BRAVE: bile flow alteration; reduction of gastric size; anatomical gut rearrangement and altered flow of nutrients; vagal manipulation and; enteric gut hormone modulation
CPAP: continuous positive airway pressure
ESS: epworth sleepiness scale
EWL: excess weight loss
LAGB: laparoscopic adjustable gastric banding
LRYGB: laparoscopic Roux-en-Y gastric bypass
LSG: laparoscopic sleeve gastrectomy
OSA: obstructive sleep apnoea
